# Phase Transformations in the High-T_c_ Superconducting Compounds, *Ba_2_RCu_3_O_7−δ_ (R = Nd, Sm, Gd, Y, Ho*, and *Er*)

**DOI:** 10.6028/jres.111.004

**Published:** 2006-02-01

**Authors:** W. Wong-Ng, L. P. Cook, H. B. Su, M. D. Vaudin, C. K. Chiang, D. R. Welch, E. R. Fuller, Z. Yang, L. H. Bennett

**Affiliations:** National Institute of Standards and Technology, Gaithersburg, MD 20899-0001; Beckman Institute, California Institute of Technology, Pasadena, CA 91125; National Institute of Standards and Technology, Gaithersburg, MD 20899-0001; Department of Materials Science, Brookhaven National Laboratory, Upton, NY 11973; National Institute of Standards and Technology, Gaithersburg, MD 20899-0001

**Keywords:** ac magnetic susceptibility, orthorhombic/tetragonal transformation, oxygen ordering, phase transformation of Ba_2_RCu_3_O_7−δ_ (R=lanthanides), strain effect on phase transition, transition temperature

## Abstract

The phase transformation between the orthorhombic and tetragonal structures of six high-*T*_c_ superconductors, Ba_2_RCu_3_O_7−_*_δ_*, where R = Nd, Sm, Gd, Y, Ho, and Er, and *δ* = 0 to 1, has been investigated using techniques of x-ray diffraction, differential thermal analysis/thermogravimetric analysis (DTA/TGA) and electron diffraction. The transformation from the oxygen-rich orthorhombic phase to the oxygen-deficient tetragonal phase involves two orthorhombic phases. A superlattice cell caused by oxygen ordering, with *a*′ = 2a, was observed for materials with smaller ionic radius (Y, Ho, and Er). For the larger lanthanide samples (Nd, Sm, and Gd), the *a*′ = 2a type superlattice cell was not observed.

The structural phase transition temperatures, oxygen stoichiometry and characteristics of the *T*_c_ plateaus appear to correlate with the ionic radius, which varies based on the number of f electrons. Lanthanide elements with a smaller ionic radius stabilize the orthorhombic phase to higher temperatures and lower oxygen content. Also, the superconducting temperature is less sensitive to the oxygen content for materials with smaller ionic radius. The trend of dependence of the phase transformation temperature on ionic radius across the lanthanide series can be explained using a quasi-chemical approximation (QCA) whereby the strain effect plays an important role on the order-disorder transition due to the effect of oxygen content on the CuO chain sites.

## 1. Introduction

Recent advances in coated conductor science and engineering have brought commercial high-*T*_c_ superconductor technology closer to reality [1]. It is now likely that many potential large-scale industrial applications will soon be realized. Three state-of-the-art technologies for producing textured coated conductors show promise: Ion Beam Assisted Deposition (IBAD) [2–5], Rolling Assisted Bi-axially Textured Substrate (RABiTS) deposition [6–11], and Inclined Substrate Deposition (ISD) technique [12,13]. Good quality textured conductors, which are based on the Ba_2_YCu_3_O_7−_*_δ_* superconductor, have been successfully produced with both film deposition and open-air solution techniques [14–22]. To further optimize the superconducting properties of long-length coated conductors for practical applications, recent research has also included the use of lanthanide-substituted variants, Ba_2_RCu_3_O_7−_*_δ_* (R=lanthanides with stable 3+ oxidation state). Phase equilibrium research pertaining to Ba_2_RCu_3_O_7−_*_δ_*, including a thorough understanding of phase transition phenomena, is therefore important for processing.

The progressive reduction in size of the lanthanide, which is known as the lanthanide contraction, allows systematic study of the trend of crystal chemistry, solid solution formation, and phase equilibria in the systems BaO-R_2_O_3_-CuO_x_ as a function of the size of lanthanide ion, R^3+^ [23–29]. Numerous investigations pertaining to the crystal chemistry, and the effect of oxygen stoichiometry on properties of Ba_2_RCu_3_O_7−_*_δ_* have been reported [30–35]. The present paper is part of our continuing effort to understand the effect of lanthanide substitution on the properties and processing parameters of the high-*T*_c_ superconductors BaR_2_Cu_3_O_7−_*_δ_* [23–29,36–39]. Since our preliminary reports [36–38] on the structural phase transitions of Ba_2_RCu_3_O_7−_*_δ_*, for R = Sm, Gd, Er, Y, and Ho, we have completed studies of the Nd series, including additional characterization of some of these materials using electron diffraction techniques. We have also improved our understanding of the phase transition in Ba_2_RCu_3_O_7−_*_δ_* as related to the size of ionic radius of R^3+^ by using a quasi-chemical approximation to describe the effect of oxygen order-disorder [40].

After annealing the Ba_2_RCu_3_O_7−_*_δ_* compounds at various temperatures and then rapidly quenching to liquid nitrogen temperature, we have made several observations concerning the phase transitions in these materials. Most interesting is the presence of *T*_c_ plateaus (annealing temperature ranges over which *T*_c_ remains approximately constant, even as the oxygen content changes). Examples are given of the existence of a 90 K and a 60 K *T*_c_ plateau in the Y, Ho, and Er materials [36–38,41–42]. Although there are extensive literature reports [43–49] describing the experimental observations or theoretical predictions of a single phase 60 K material in the Y system, very little information has previously been reported describing the lanthanide-substituted materials. Furthermore, most of the reported Y materials were prepared using processing routes different from our quenching methods [46–49].

## 2. Experimental

Since Nd_2_O_3_ reacts with atmospheric moisture to form Nd(OH)_3_, the Nd_2_O_3_ powder was heat-treated at 600 °C overnight prior to sample preparation. A single phase master batch of Ba_2_NdCu_3_O_7−_*_δ_* was prepared from a stoichiometric mixture of CuO, Nd_2_O_3_ and BaCO_3_. Before firing and annealing, the powder was pressed into pellets and placed on MgO single crystals. The pellets were reground and annealed several times until the presence of a single-phase material was confirmed by x-ray powder diffraction analysis. Annealing was carried out at temperatures of 850 °C, 870 °C, and 900 °C each in air for 1 d, followed by firing in air at 940 °C for 2 d. Analysis by scanning electron microscopy and energy dispersive x-ray analysis (SEM/EDS) showed absence of substitution of magnesium in these samples.

**Fig. 1:**
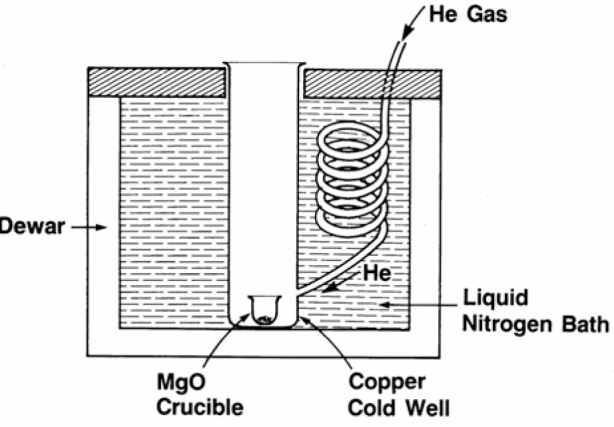
An experimental setup showing a liquid nitrogen cooled copper cold well into which liquid helium was rapidly flowing through as an annealed sample that was contained in a MgO crucible was quenched.

A total of 10 barium-neodymium-copper-oxide samples were prepared for this investigation from the single-phase master batch. Batches of the other Ba_2_RCu_3_O_7−_*_δ_* phases were prepared in similar fashion, using the appropriate lanthanide oxides [36–38]. To investigate the phase transitions, specimens weighing about 200 mg to 500 mg were annealed in an MgO crucible for up to 2 d in air at temperatures between 400 °C and 900 °C. The temperature was measured using a Pt/Pt10Rh thermocouple calibrated against the melting point of gold. Temperature of the Pt-wound resistance furnace used in these experiments was controlled to ± 2 °C by using a Wheatstone bridge type controller. After annealing, the samples were quenched into a liquid nitrogen-cooled copper cold well, through which liquid nitrogen-cooled helium gas was passed at a rapid rate (Fig. 1). Rapid cooling under these conditions prevented the oxygen gain that would normally occur for samples cooled in air.

Samples studied by electron microscopy are listed in Table 1, together with their processing conditions and oxygen content (all were quenched in liquid nitrogen-cooled helium). Specimens for electron microscopy were prepared by crushing small pieces of the sample material and then following one of two procedures: either the powder was dispersed in ethanol and drops of the liquid were placed on a carbon-coated copper grid, or a grid was scooped through the powder to avoid possible reactions with the alcohol. These two techniques produced samples with no detectable differences between them. Conventional electron microscopy and electron diffraction were carried out at 120 keV and 300 keV, and exposure times up to 50 s were used to record the faint superlattice diffraction spots.

The oxygen content of the Ba_2_RCu_3_O_7−_*_δ_* compounds was determined by thermogravimetric analysis (TGA) from measurements of the weight change as a function of temperature in both air and in an oxygen atmosphere. An MgO sample holder was used for the yttrium compound and a platinum sample holder was used for the other four samples, since the temperatures of the TGA curves were below those at which any reactions with platinum were observed. The ground powder was heated from 50 °C to 850 °C at a rate of 2 °C per min to measure the change of weight, and thereby oxygen uptake and loss. The maximum observed weight was assumed to correspond to full oxygenation of seven oxygen atoms, or Ba_2_RCu_3_O_7_. The weight loss at a given temperature was then used to compute the oxygen content. The correspondence between oxygen content and quenching temperatures was thus established. Due to the difficulty of applying a precise correction for the buoyancy effect, oxygen content established in this way may have an estimated relative uncertainty of ± 5 % (*k*=2).

Digital x-ray data were collected at room temperature on a computer-controlled powder diffractometer equipped with a focusing graphite crystal monochromator and a theta-compensating slit. Copper radiation (CuKα_1_, *λ* = 1.5405981 Å [50]) was employed for all studies. Two certified *d*-spacing standards: silicon, SRM640b [51], and fluorophlogopite, SRM675 [52] were used as internal standards for calibration [53]. Sample preparation, mounting methods and data processing followed those described by McMurdie et al. [54].

The flux exclusion of these powders was studied by using a computerized ac magnetometer. A sample powder of 10 mg to 20 mg was packed in a small, non-magnetic holder and mounted on a stage containing a calibrated silicon diode thermometer. The ac susceptibility was measured as a function of temperature from 300 K to 20 K for most samples in a Hartshorn type bridge circuit at a frequency of 1.68 kHz. The magnitude of the applied ac field was about 0.5 × 10^−4^ T. The relative Meissner effect was detected by observing the real part of the signal arising from the diamagnetism of the sample. We define the superconducting onset temperature, *T*_CO_, as the temperature at which the ac susceptibility deviates from the near zero value of the normal state.

## 3. Results and Discussion

**Fig. 2:**
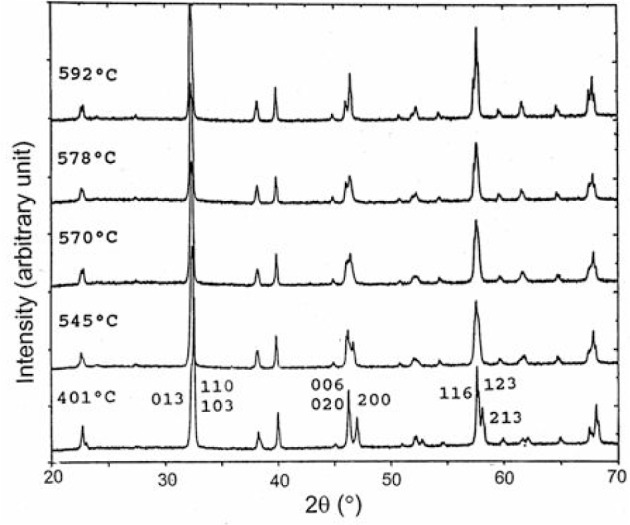
X-ray diffraction patterns (CuKα_1_) from Ba_2_NdCu_3_O_7−_*_δ_* samples quenched from 401 °C, 545 °C, 570 °C, 578 °C, and 592 °C, showing progressive changes of peak shapes. Selected Miller indices (hkl values) of the diffraction peaks for the sample quenched from 401 °C are indicated.

The x-ray spectra of Ba_2_RCu_3_O_7−_*_δ_* (*δ* from 0 to 1) are, in general, similar to the yttrium analogs [24]. The progressive changes of shape and the indexing of peaks in the five main regions of the Bragg angle, 2*θ*, around 32° to 33°, 38° to 39°, 45° to 49°, 57° to 60°, and 68° to 70° reveal the crystallographic phase transition from orthorhombic to tetragonal for all compounds. Figure 2 illustrates the diffraction patterns of the Ba_2_NdCu_3_O_7−_*_δ_* samples quenched from various temperatures at 401 °C, 545 °C, 570 °C, 578 °C and 592 °C. These diffraction patterns demonstrate a gradual reduction in orthorhombicity as evidenced, for example, by the peak shape changes for the 200 and 020 reflections at 46° to 48° 2*θ* and the 213 and 123 reflections at 57° to 59° 2*θ*.

### 3.1 Lattice Parameters

Using the least-squares refinement results [55], the transition from orthorhombic to tetragonal symmetry was estimated to take place between 570 °C and 578 °C. No doubling of cell parameters along either the *a* or *b* axes has been observed for Ba_2_NdCu_3_O_7−_*_δ_* by x-ray powder diffraction. Based on a similar analysis, the structural transition temperature for the other lanthanide analogues were found to be as follows: Sm: 625 °C to 650 °C, Gd: 650 °C to 660 °C, Y: 708 °C to 720 °C, Ho: 748 °C to 761 °C, and Er: 750 °C to 770 °C [36–39,41–49]. The tetragonal-orthorhombic structural transition temperatures are summarized in Table 2 along with ionic radii of the lanthanide ions, R^3+^ [56] and the estimated oxygen composition.

**Fig. 3:**
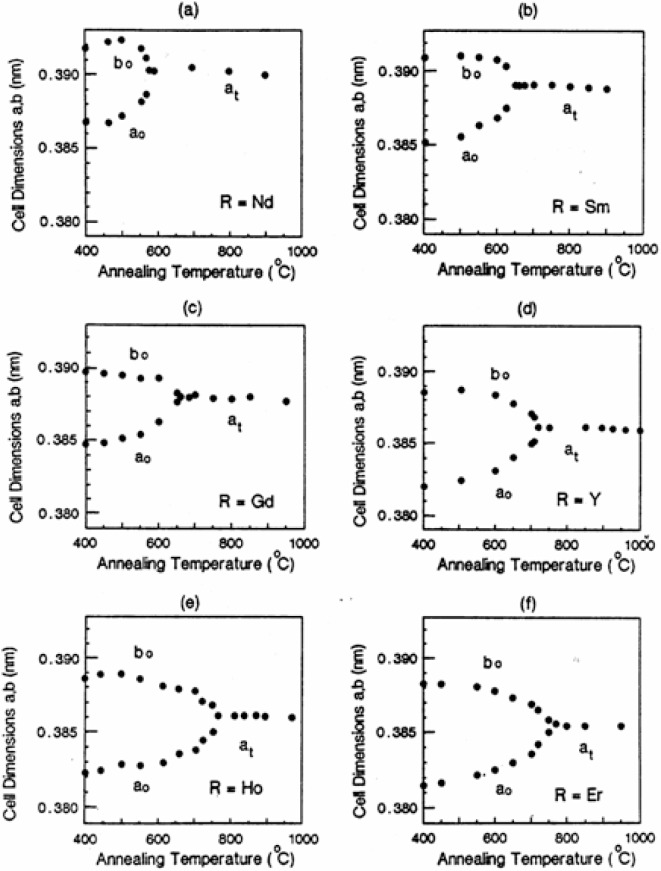
Crystallographic dependence of the *a* and *b* axes of Ba_2_RCu_3_O_7−_*_δ_* on the annealing temperature. Lanthanide ion R: (a) Nd (b) Sm (c) Gd (d) Y (e) Ho, and (f) Er.

Table 3 gives the cell parameters of Ba_2_NdCu_3_O_7−_*_δ_* calculated from the x-ray patterns. Figures 3(a) to 3(f) depict the convergence of the *a* and *b* axis dimensions as the annealing temperature rises for these six compounds. While the merging curves exhibit similar shape and form, the different positions of the convergence of these curves can be related to the size of the lanthanide ion R^3+^. The *a* and *b* axis dimensions (*a*_o_, *b*_o_) in the orthorhombic structure and the *a* axis dimension (*a*_t_) in the tetragonal structure remain the largest for Ba^2^NdCu_3_O_7−_*_δ_* across the entire annealed temperature range. Correspondingly, those of Ba_2_ErCu_3_O_7−_*_δ_* are the smallest.

**Fig. 4:**
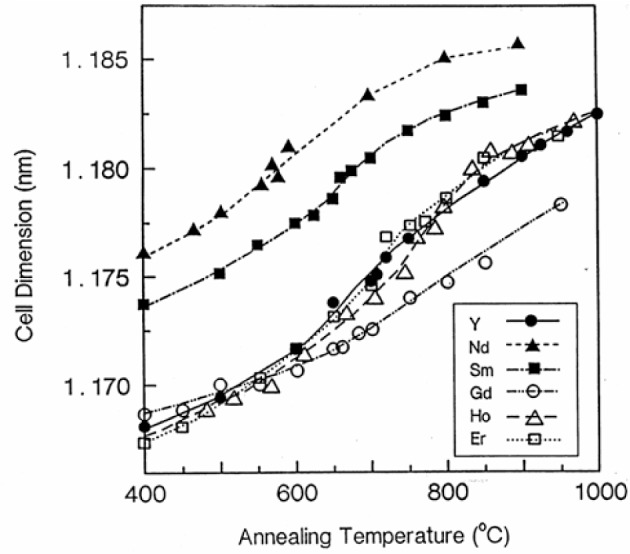
The *c* axis cell parameter as a function of annealing temperature for Ba_2_RCu_3_O_7−_*_δ_*, with R = Nd, Sm, Gd, Y, Ho, and Er.

**Fig. 5:**
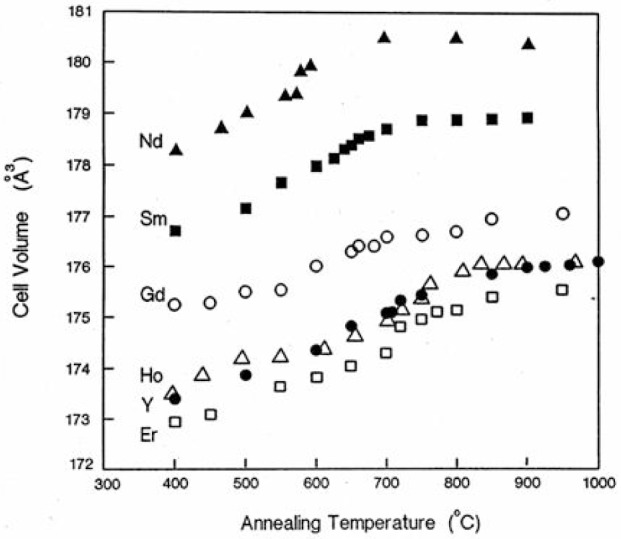
Unit cell volume as a function of annealing temperature for Ba_2_RCu_3_O_7−_*_δ_*, with R = Nd, Sm, Gd, Y, Ho, and Er.

The variation with annealing temperature of the *c* axis cell dimension and the cell volume of these six compounds is illustrated in Fig. 4. Although these curves show, in general, the expected trend of increasing value as the temperature increases, the Ba_2_GdCu_3_O_7−_*_δ_* compounds behave somewhat differently. For example, the *c*-dimension did not fall in the expected order relative to the other compounds. By analogy with the yttrium system, the elongations along the *c* axis of these compounds are considered to be due to the increased number of oxygen vacancies. At 400 °C the relative *c* axis cell dimensions of these compounds are as expected: *c*_Nd_ > *c*_Sm_ > *c*_Gd_ > *c*_Y_ ~ *c*_Ho_ > *c*_Er_. At higher temperature the trend alters and becomes *c*_Nd_ > *c*_Sm_ > *c*_Ho_ > *c*_Y_ > *c*_Er_ > *c*_Gd_. The volume plots in Fig. 5 illustrate the expected trends in volume expansion as the annealing temperatures increase, namely, *V*_Nd_ > *V*_Sm_ > *V*_Gd_ > *V*_Y_ ~ *V*_Ho_ > *V*_Er_.

**Fig. 6:**
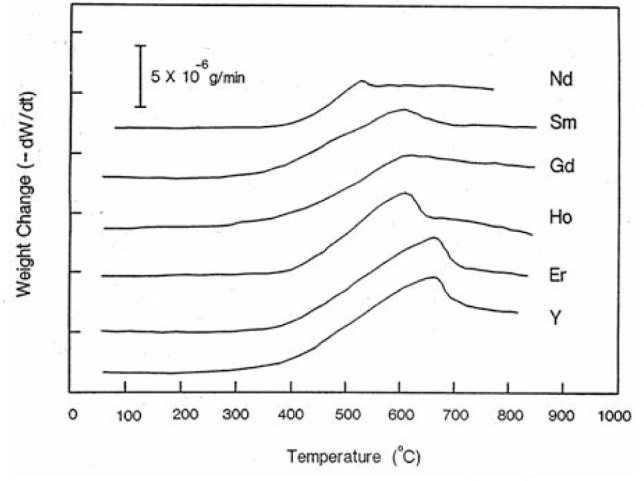
Thermogravimetric analysis of Ba_2_RCu_3_O_7−_*_δ_* showing the slope of the heating curve.

Figure 6 shows the first derivative of the TGA curves against temperature for the six compounds. While the weight change curves (not shown) are continuous, the first derivatives show relatively abrupt changes in slope. The temperature at which this abrupt change in temperature takes place can be considered as due to a phase transition, presumably the orthorhombic/tetragonal transition, and the trend of this behavior therefore parallels that listed in Table 2, namely, the lanthanide elements with a smaller ionic radius stabilize the orthorhombic phase to higher temperatures.

### 3.2 Chain-Oxygen Order-Disorder Transition

**Fig. 7:**
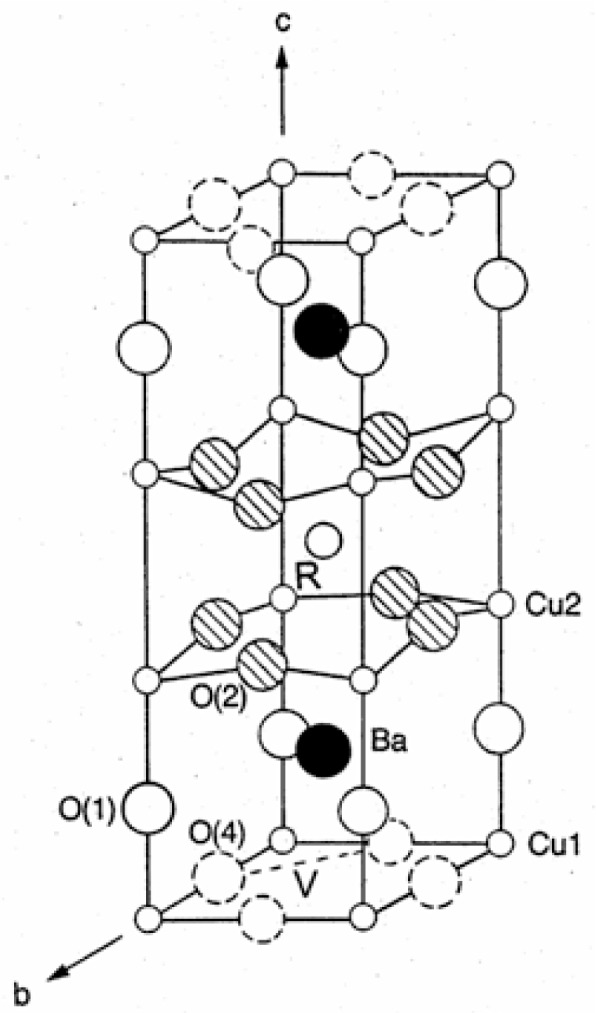
Crystal structure and atom labels for Ba_2_RCu_3_O_7−_*_δ_*. The repulsion energy, *v*, between the oxygen atoms on two sublattice sites is represented as “*v*”.

**Fig. 8:**
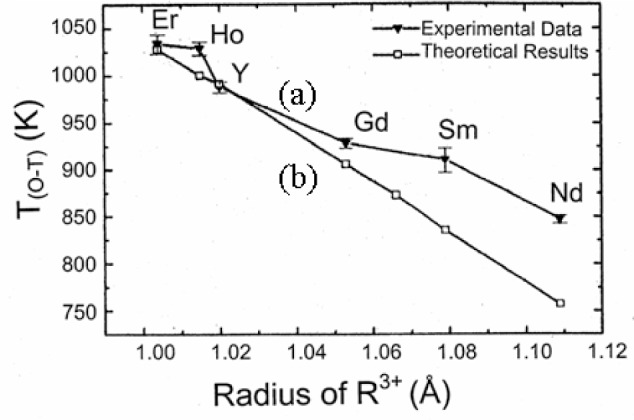
Chain-oxygen order-disorder experimental (curve (a)) and theoretical (curve (b) [40]) phase transition temperatures vs. Shannon’s ionic radius of R^3+^(VIII-coordination) [55]. The theoretical data are calculated by using the transition temperature of Ba_2_YCu_3_O_7−_*_δ_* as a reference.

Figure 7 shows the structure of Ba_2_RCu_3_O_7−_*_δ_* with the labeling of atoms and the oxygen sublattice site. Curve (a) of Fig. 8 shows a plot of these experimental transition temperatures as a function of the ionic radius of the R^3+^ ions. An obvious trend is observed. Lanthanide elements of smaller ionic size stabilize the orthorhombic phase to a higher temperature as well as to lower oxygen content. This trend can be understood in terms of order-disorder theory.

Theoretical studies aimed at understanding the phase transformation in the Ba_2_YCu_3_O_7−_*_δ_* system have been carried out extensively by Wille et al. [44], Bakker et al. [57], and Herman [58]. Recently, the similar approach has been applied by Su et al. to the lanthanide-substituted systems [40]. In brief, the formation energies of Frenkel pair defects as a function of volumetric strain for Ba_2_RCu_3_O_7−_*_δ_*, and for Ba_2_RCu_3_O_7_ under hydrostatic pressure were calculated. These theoretical calculations show good agreement with experimental observations in that increased pressure favors ordering of the CuO chains. For example, the Frenkel pair formation energy indeed increases significantly (around −0.25 eV/0.01 volumetric strain) under compression.

Based on a quasi-chemical approach (QCA) by Bakker et al. [57], the orthorhombic/tetragonal transition temperatures for Ba_2_RCu_3_O_7−_*_δ_* have been computed by scaling the effective oxygen-oxygen short-range repulsive energy in the CuO chain. At a given temperature *T*, a simplified relation [Eq. (1)] can be obtained to express the oxygen-oxygen repulsion energy, *v*, on two sublattice sites α and β, as a function of the long range parameter (*S*), the short range order expressed by the fraction (*p*) of near-neighbor pair sites occupied by oxygen-oxygen pairs, and the fractional site occupancy averaged over both sublattices (c).

$$v/kT=\ln\left(\frac{\left(c\left(1+S\right)-p\right)\left(c\left(1-S\right)-p\right)}{p\left(1-2c+p\right)}\right).$$ (1)

The fraction *p* is equal to *N*_00_/4*N*, where *N* is the number of sites on each of the sublattice, and *N*_00_ is the number of oxygen-oxygen near neighbor pairs. In Eq. (1)*k* is the Boltzmann constant. The long-range order parameter is defined such that the fractional site occupancy of oxygen on one of the two sublattice site β is *c*(1+*S*), while that on sublattice α is *c*(1−*S*). In Ba_2_YCu_3_O_7−_*_δ_*, *c* is assumed to be 0.5 when *δ* is zero, and *δ* = 1 − 2*c*. The order-disorder transition temperature (orthorhombic to tetragonal), *T*_O−_*_T_*, is therefore related to the oxygen-oxygen repulsion energy, *v*, on the two sublattice sites and the value of the average site occupancy *c* by

$$kT_{Ο-T}=v\ln\left\{16\left(1-c\right)/\left[1-4{\left(1-2c\right)}^{2}\right]\right\}.$$ (2)

The trend of dependence of the ionic radius across the lanthanide series using the QCA is summarized in Fig. 8. The upper curve (a) represents the experimental data taken from this work while the lower one (b) represents the theoretical results using the current data (i.e., experimental phase transition temperatures) [40]. The theoretical data are calculated by using the transition temperature of Ba_2_YCu_3_O_7−_*_δ_* as a reference (transition takes place at an oxygen content of 6.5). The calculated results agree with experimental data in that the larger the ionic size of R, the lower the orthorhombic/tetragonal phase transition temperature. The observable difference in these two curves is partly because the orthorhombic to tetragonal transition in the R-systems takes place at an oxygen content different from that of the reference Y-system, namely, 6.4 (Er) to 6.83 of Nd. The formation energy of Frenkel pairs is altered because of the difference of oxygen content (which affect lattice parameters and atomic positions). This formation energy of Frenkel pairs decreases as anisotropy in the *ab* plane [(*b*−*a*)/*a*] decreases.

From a simple point of view, if an assumption is made that the orthorhombic phase (absence of oxygen on the *a* axis) is favored at lower temperatures, then as the size of the lanthanide ion decreases, so does the distance between neighboring basal oxygens; the repulsion energy, *v* (Fig. 7), between these oxygens increases correspondingly. The transformation temperature, according to Eq. (2), is directly proportional to the repulsion energy, and is therefore higher the smaller the size of R.

### 3.3 *T*_c_ Dependence of Oxygen Content

**Fig. 9:**
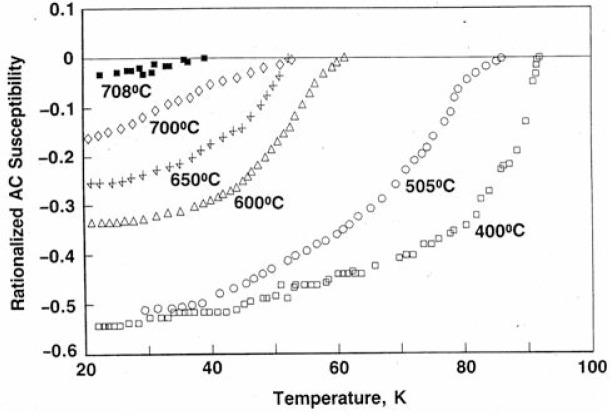
A plot of the rationalized ac susceptibility as a function of temperature. The annealing temperatures are indicated. All samples were fully packed fine powders. Samples annealed at 750 °C and above did not exhibit any flux exclusion.

Figure 9 shows a typical plot of the rationalized ac susceptibility of Ba_2_RCu_3_O_7−_*_x_* [31] as a function of temperature. The annealing temperatures are indicated from 400 °C to 708 °C. A bulk sample exhibiting 100 % flux exclusion would have an ac susceptibility of −1 (dimensionless). Finnemore et al. [59] has shown that 100 % flux exclusion is not expected for fine powders of a completely superconducting material. It is thus not possible to determine exactly the fraction of the sample that is superconducting from the curves of Fig. 9. However, these curves have the approximate shape and magnitude expected for a ratio of particle diameter to superconducting penetration depth between 2 and 10 [59]. The decrease in the magnitude of the flux exclusion seen in Fig. 9 for the samples annealed at higher temperatures can be due either to a decrease in the fraction of the materials that is superconducting or to an increase in the superconducting penetration depth, or likely, to a combination of both.

**Fig. 10:**
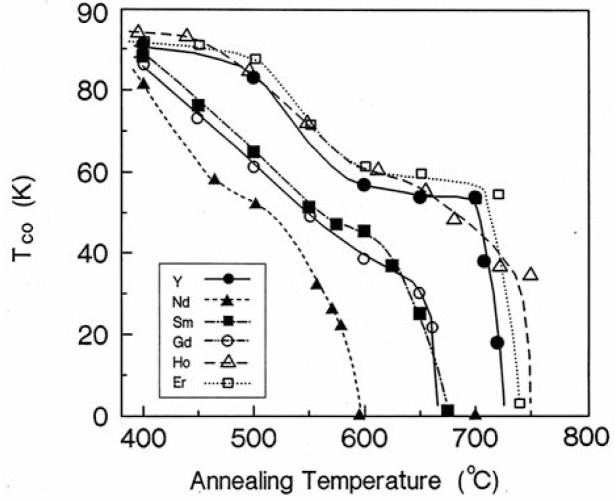
Dependence of the superconducting transition temperature, as determined by ac magnetic susceptibility, on the annealing temperature in Ba_2_RCu_3_O_7−_*_δ_*, with R = Nd, Sm, Gd, Y, Ho, and Er.

Figure 10 shows plots of the superconducting temperature onset, *T*_CO_, obtained from flux exclusion measurements as a function of the annealing temperature for all six compounds. Two apparent plateaus in *T*_CO_ were observed for the materials with yttrium, holmium and erbium substitution: one at 83 K to 92 K and the other at 58 K to 60 K. Narrower and somewhat lower plateaus were detected for the gadolinium, samarium and neodymium compounds. Although, in general, the orthorhombic structure is superconducting whereas the tetragonal is not, this structure correlation does not appear to be exact. For example, the tetragonal yttrium material annealed at 719 °C is superconducting, whereas the orthorhombic Er compound annealed at 750 °C is a non-superconductor. Furthermore, despite reports from literature that even in the Ba_2_YCu_3_O_7−_*_δ_* system alone, one can achieve plateau features varying from broad plateaus at 60 K and 90 K to complete absence of plateaus depending on how samples were prepared [60], our samples were all prepared under similar conditions, therefore it is possible that one should be able to correlate the features of these plateaus with size of R.

**Fig. 11:**
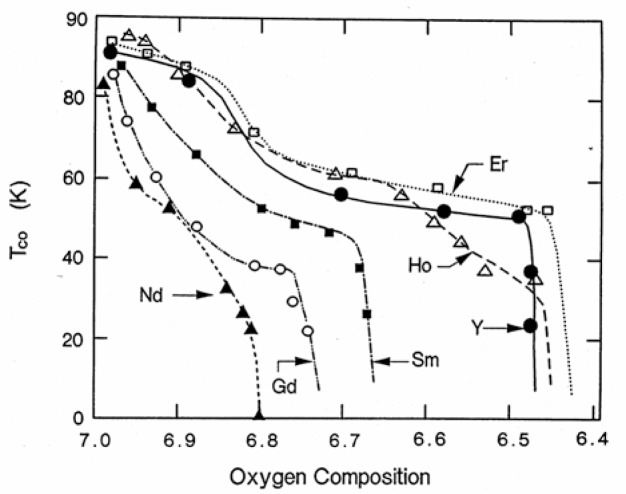
Dependence of superconducting transition temperature, as determined by ac magnetic susceptibility, on the oxygen content in Ba_2_RCu_3_O_7−_*_δ_*, with R = Nd, Sm, Gd, Y, Ho and Er.

Figure 11 depicts the oxygen content dependence of the transition temperatures of these six compounds as derived from thermogravitmetric analysis/differential thermal analysis (TGA/DTA) data. It is noteworthy that a correlation exists between the size of the R^3+^ ion and both the phase transition temperatures (or oxygen compositions) and *T*_c_ values for these plateaus, as is summarized in Table 4. Compounds with a smaller size lanthanide 3+ ion have a tendency to have both a wider 90 K plateau in *T*_c_ and a wider and relatively higher *T*_c_ in the 50 K to 60 K range. The Nd, Sm and Gd samples behave otherwise. They lack any obvious 90 K plateau, and they also play a narrow low *T*_c_ plateau (55 K, 52 K and 38 K, respectively). The observed trends appear to differentiate the early and later members of the lanthanide series.

### 3.4 Structural Features of Ba_2_RCu_3_O_7−_*_δ_*

The behavior of the curves of *T*_c_ versus annealing temperature (Fig. 10) and *T*_c_ versus oxygen content (Fig. 11) suggests the presence of more than one structural phase. X-ray results indicated both to be orthorhombic and they are designated here as O(A) and O(B). Other studies such as that of Cava et al. [41] have suggested that the second plateau region indicates the presence of a second orthorhombic phase. The presence of an O(B) phase in the yttrium sample has been confirmed by electron diffraction studies, which gave information about the degree to which ordering of the oxygen ions had occurred in the specimens. Diffraction patterns from the yttrium specimens (Y6.85, Y6.63, and Y6.55, as defined in Table 1) contained elongated superlattice reflections (streaks) lying along the [100] direction and centered on positions ***g*** + ½00 in reciprocal space (where ***g*** is a reciprocal lattice vector of the conventional orthorhombic structure).

A typical image of a crystallite in the yttrium specimen, Y6.55, together with a diffraction pattern from the crystallite, are shown in Fig. 12. The image, Fig. 12(a), shows an approximately regular arrangement of twins. From the diffraction pattern, Fig. 12(b), we can determine that the habit plane of the twins is (110), as expected, and that the orthorhombicity (*b*/*a*) of the material is 1.01, which agrees with the value of 1.008 obtained previously [37]. Diffuse scattering spots were also observed in the diffraction patterns, as indicated by the arrows. Despite the twinning, by a careful examination of the diffraction pattern far from the transmitted beam where the splitting of the orthorhombic matrix spots was greatest, it was possible to determine that the superlattice streaks were at ***g*** + ½00 and not ***g*** + 0½0. This corresponds to a doubling of the unit cell dimension along the *a* axis, suggesting that the oxygen atoms of every other CuO chain, running along the *b* axis, are removed. These results agree with those reported by Alario-Franco et al. [42], who observed diffuse scattering in a Ba_2_YCu_3_O_7−_*_δ_*, *δ* = 0.5 sample. For the yttrium specimen, Y6.85, the streaks at ½00 were particularly faint and long, as is expected as the oxygen content increases. In all cases where superlattice streaks were observed, there was variation in both the intensity and length of the streaks between different grains from the same specimen, indicating that the oxygen content was not constant throughout the specimen and that the diffusion of oxygen through the lattice is slow at these temperatures. However, on average, the lengths of the streaks for specimens Y6.63 and Y6.55 corresponded to short range order on the 5 nm to10 nm (50 Å to100 Å) scale.

Although short range ordering was observed in the Y sample using electron diffraction, long range ordering was not observed using either electron diffraction or powder x-ray diffraction. Neutron scattering studies on a 60 K yttrium material annealed at 640 °C also showed no evidence of long range ordering. The results agreed with those of the x-ray powder diffraction and indicate the absence of any doubling of the cell parameters along either the *a* or *b* axis. The nature of supercell ordering in Y-123 Ba_2_YCu_3_O_7−_*_δ_* have been studied extensively by Beyers et al [61], Zeiske et al. [62], and Ourmazd and Spence [63]. De Fontaine et al. [64] suggested that this supercell is stabilized at low temperature. It is now generally agreed that the degree of plateau behavior of Ba_2_YCu_3_O_7−_*_δ_* depends on the degree to which the ordered 2*a* × *b* × *c* supercell is stabilized [65].

The gadolinium specimen was unique in that there was very little twinning in the crystallites studied; all the other lanthanide specimens were twinned. Diffraction patterns from the neodymium, samarium and gadolinium specimens (Nd6.90, Sm6.75 and Gd6.79) showed no evidence of superlattice formation of the type corresponding to the doubling of the unit cell dimension along the *a* axis. It is conceivable that the O(B) phase in compounds with smaller size R, has a doubling of the unit cell dimension along the *a* axis, corresponding to the absence of oxygen in every other Cu-O chain along the *b* axis. For the larger size R, the compositions with lower *T*_c_ plateau that we investigated correspond to those deviating significantly from the oxygen content of 6.50. We postulate that these lower *T*_c_ plateau regions of 55 K, 52 K, and 38 K in the Nd, Sm and Gd samples may result from another orthorhombic phase with a superlattice cell of the type corresponding to different oxygen vacancies. A superlattice cell other than the type with *a*′ = 2*a* has also been reported. For example, Alario-Franco et al. [42] reported a superlattice type in the Y compound corresponding to an oxygen content of 6.85; this superlattice can be indexed on a unit cell of $2\sqrt{2}a_{c}\times 2\sqrt{2}a_{c}\times 3a_{c}$, where *a*_c_ is the basic cubic perovskite cell dimension.

**Fig. 13:**
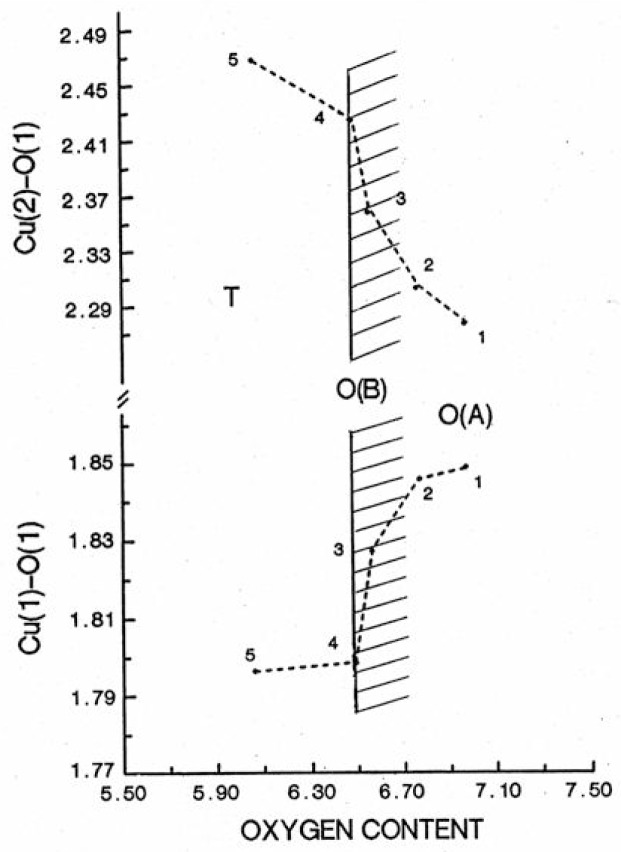
Illustration of the bond lengths, Cu(1)-O(1) and Cu(2)-O(1), as a function of oxygen content in the Ba_2_YCu_3_O_7−_*_δ_* samples.

Oxygen stoichiometry is an important parameter affecting the *T*_c_ depression and the presence of *T*_c_ plateaus. The CuO chain in the basal plane and the CuO plane [66–69] can be considered as an interacting electronic unit. Tokura et al. [70] and Cava et al. [41,71] have suggested that the chains function as a charge reservoir which controls the electron density on the Cu-O planes. By applying this concept to our samples, when oxygen atoms are removed from the chains, the electrons which were bound to them are transferred to the lowest unoccupied energy level. When the total oxygen content is near 7, the *T*_c_ value of 90 K is due to the coupling of the chains and the planes. As illustrated in Fig. 13, when oxygen atoms are removed from the chains, the chain copper-bridging oxygen distance, Cu(1)-O(1), becomes shorter and the plane copper-bridging oxygen distance, Cu(2)-O(1), becomes longer, with the result that the chains and the planes become decoupled. The distance Cu(1)-O(1) first shortens gradually in the orthorhombic O(A) region, then changes rather sharply in region B [36,72]. In the tetragonal region it resumes a much smaller slope. The Cu(2)-O(1) bond elongates in a similar way in these materials. It appears that the decoupling effect of the chains and the planes takes place much more dramatically in region O(B). The observed superconductivity at the lower *T*_c_ regions of 60 K, 60 K, 58 K, 38 K, 52 K and 55 K in the Er, Ho, Y, Gd, Sm and Gd compounds, respectively, is probably due to sufficient decoupling of the chain-plane unit, and the different *T*_c_ are intrinsic values of the plane. When the capacity of the CuO chain-reservoirs to hold charge is exhausted, charge is transferred from the chains to planes, and superconductivity disappears. This charge transfer is equivalent to a decrease in the hole concentration in the planes [41,70].

## 4. Conclusions

The structural phase transformation that occurs in the high-*T*_c_ ceramic superconductors is of considerable importance in the processing of these materials. We have found that the temperature of this structural phase transition, its oxygen stoichiometry, and characteristics of the associated *T*_c_ plateaus follow a trend depending on the ionic radius of the lanthanide ions. Lanthanide elements with a smaller ionic size stabilize the orthorhombic phase to higher temperatures, or lower oxygen content. The superconducting temperature is less sensitive to the oxygen content for materials with smaller ionic radii.

Electron microscopy studies indicated that the O(B) phase in the Y compound has a doubling of the unit cell dimension along the *a* axis, corresponding to the absence of oxygen in every other Cu-O chain along the *b* axis. While not yet investigated, this type of superlattice can probably also be found in Ba_2_RCu_3_O_7−_*_δ_* compounds with smaller size R. This is most likely short-range ordering as both the x-ray and neutron diffraction data indicate a lack of evidence for a long-range ordering of oxygen in samples quenched around the “lower” *T*_c_ plateau region. For the lanthanide samples with larger size of R (i.e., Nd, Sm, and Gd), the lower *T*_c_ plateau is postulated as corresponding to a different superlattice resulting from different oxygen stoichiometry.

Our study also illustrates the importance of strain effects on the orthorhombic/tetragonal phase transition in the Ba_2_RCu_3_O_7−_*_δ_* compounds. The formation energy of Frenkel pair defects as a function of volumetric strain for Ba_2_RCu_3_O_7−_*_δ_*, and for Ba_2_YCu_3_O_7_ under hydrostatic pressure show good agreement with experimental observations that pressure favors ordering of the CuO chains. For example, the Frenkel pair formation energy indeed increases significantly (around −0.25 eV/0.01 volumetric strain) under compression. Based on a quasi-chemical approach, the orthorhombic/tetragonal transition temperatures for Ba_2_RCu_3_O_7−_*_δ_* have been computed by scaling the effective oxygen-oxygen short-range repulsive energy in the CuO chain using the Frenkel pair formation energy. The calculated results agree with experimental data in that the larger the ionic size of the lanthanide, the lower the orthorhombic/tetragonal phase transition temperature.

## Figures and Tables

**Fig. 12(a):**
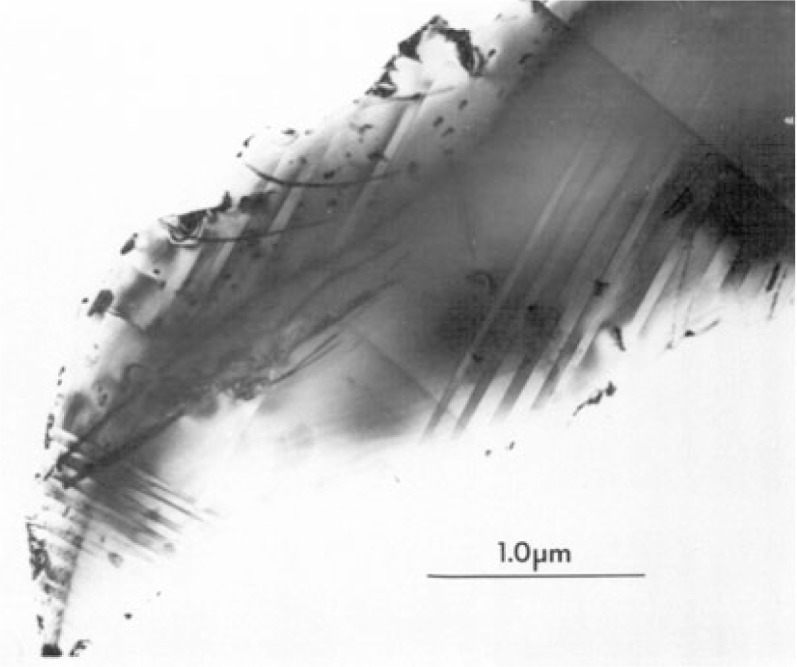
TEM micrograph of Ba_2_YCu_3_O_7−_*_δ_* grain annealed at 675 °C for 21 h showing typical twin boundary arrangement.

**Fig. 12(b):**
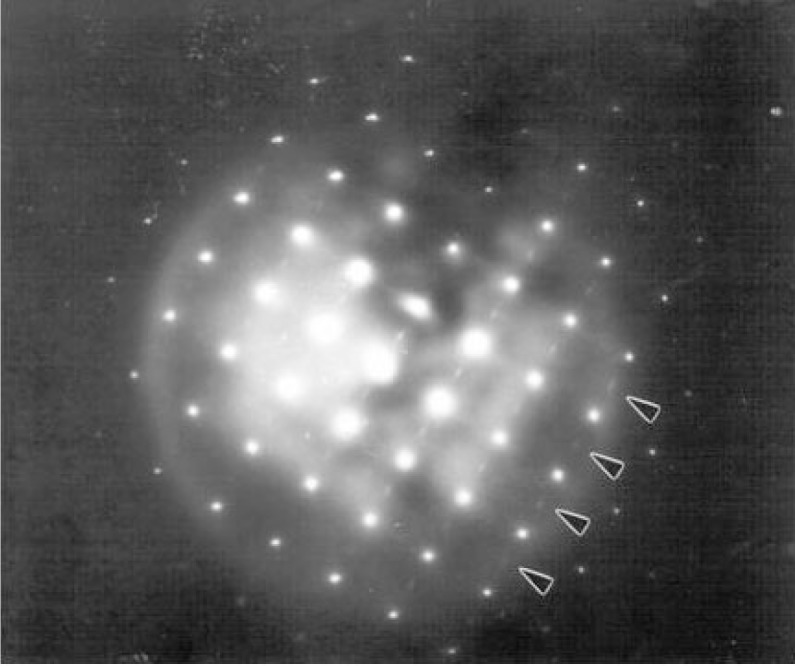
Electron diffraction pattern from the grain shown in Fig. 12(a). The diffuse spots (indicated by arrows) correspond to a doubling of the *a* axis (a superlattice indexable with a cell of *a*′ ≈ 2*a*). Twinning is demonstrated by the splitting of the diffraction spots.

**Table 1:** Samples investigated by electron diffraction techniques in the transmission electron microscope

Sample notation	Chemical formula	Annealing temperature	Time	Oxygen content (7−*δ*)
Nd6.90	Ba_2_NdCu_3_O_7−_*_δ_*	620 °C	20 h	6.90
Sm6.75	Ba_2_SmCu_3_O_7−_*_δ_*	580 °C	40 h	6.75
Gd6.79	Ba_2_GdCu_3_O_7−_*_δ_*	620 °C	20 h	6.79
Y6.85	Ba_2_YCu_3_O_7−_*_δ_*	502 °C	36 h	6.85
Y6.63	Ba_2_YCu_3_O_7−_*_δ_*	635 °C	23 h	6.63
Y6.55	Ba_2_YCu_3_O_7−_*_δ_*	675 °C	21 h	6.55

**Table 2:** Shannon’s ionic radii [56] for R^3+^ (VIII-coordination), tetragonal orthorhombic phase transition temperature and oxygen content, (*x* = 7−*δ*), for selected Ba_2_RCu_3_O_7−_*_δ_*

Compound	Shannon’s ionic radius R^3+^ (Å) [56]	Temperature (°C)	Oxygen content
Ba_2_NdCu_3_O_7−_*_δ_*	1.109	570 to 578	6.82–6.84
Ba_2_SmCu_3_O_7−_*_δ_*	1.079	625 to 650	6.65–6.69
Ba_2_GdCu_3_O_7−_*_δ_*	1.053	650 to 660	6.75–6.76
Ba_2_YCu_3_O_7−_*_δ_*	1.019	708 to 720	6.47–6.49
Ba_2_HoCu_3_O_7−_*_δ_*	1.015	740 to 760	6.46–6.47
Ba_2_ErCu_3_O_7−_*_δ_*	1.004	750 to 770	6.38–6.41

**Table 3:** Least-squares cell parameters for Ba_2_NdCu_3_O_7−_*_δ_* as a function of quenched temperatures (°C). Number in parenthesis indicates one standard deviation from results of least-square refinements [37]

Quenched temperatures	*a*(Å)	*b*(Å)	*c*(Å)	*V*(Å^3^)
401	3.8681(11)	3.9180(3)	11.762(3)	178.26(7)
464	3.8676(14)	3.9223(8)	11.779(2)	178.69(6)
501	3.8724(7)	3.9237(12)	11.781(2)	179.00(5)
556	3.882(2)	3.9180(12)	11.792(4)	179.30(9)
570	3.8870(2)	3.9115(5)	11.797(2)	179.35(8)
578	3.9034(a)		11.799(1)	179.78(3)
592	3.9029(6)		11.8103(14)	179.90(5)
697	3.9051(4)		11.8336(11)	180.46(3)
800	3.9025(3)		11.8511(12)	180.48(3)
900	3.9000(3)		11.8564(12)	180.34(3)

**Table 4:** Characteristics of the low temperature *T*_c_ plateaus for Ba_2_RCu_3_O_7−_*_δ_* with R = Nd, Sm, Gd, Y, Ho, and Er

Lanthanide R	Approximate compositional range (*x* = 7−*δ*)	Approximate anneal temperature range in air	*T*_c_
Nd	6.83 < × < 6.88	40 °C (460 °C to 500 °C)	55 K
Sm	6.82 < × < 6.83	70 °C (550 °C to 620 °C)	52 K
Gd	6.77 < × < 6.81	70 °C (580 °C to 650 °C)	38 K
Y	6.62 < × < 6.80	100 °C (600 °C to 700 °C)	58 K
Ho	6.62 < × < 6.77	80 °C (580 °C to 660 °C)	60 K
Er	6.58 < × < 6.80	120 °C (600 °C to 720 °C)	60 K
